# The potential of short-chain fatty acid epigenetic regulation in chronic low-grade inflammation and obesity

**DOI:** 10.3389/fimmu.2024.1380476

**Published:** 2024-03-27

**Authors:** Julia Kopczyńska, Magdalena Kowalczyk

**Affiliations:** Laboratory of Lactic Acid Bacteria Biotechnology, Institute of Biochemistry and Biophysics, Polish Academy of Sciences, Warsaw, Poland

**Keywords:** short-chain fatty acids, obesity, low-grade inflammation, lipopolysaccharide, epigenetics

## Abstract

Obesity and chronic low-grade inflammation, often occurring together, significantly contribute to severe metabolic and inflammatory conditions like type 2 diabetes (T2D), cardiovascular disease (CVD), and cancer. A key player is elevated levels of gut dysbiosis-associated lipopolysaccharide (LPS), which disrupts metabolic and immune signaling leading to metabolic endotoxemia, while short-chain fatty acids (SCFAs) beneficially regulate these processes during homeostasis. SCFAs not only safeguard the gut barrier but also exert metabolic and immunomodulatory effects via G protein-coupled receptor binding and epigenetic regulation. SCFAs are emerging as potential agents to counteract dysbiosis-induced epigenetic changes, specifically targeting metabolic and inflammatory genes through DNA methylation, histone acetylation, microRNAs (miRNAs), and long non-coding RNAs (lncRNAs). To assess whether SCFAs can effectively interrupt the detrimental cascade of obesity and inflammation, this review aims to provide a comprehensive overview of the current evidence for their clinical application. The review emphasizes factors influencing SCFA production, the intricate connections between metabolism, the immune system, and the gut microbiome, and the epigenetic mechanisms regulated by SCFAs that impact metabolism and the immune system.

## 1 Introduction

Obesity and inflammation are the two main causes of chronic diseases, which are considered to be the biggest global health issue nowadays (1). The long-term effects of a constant inflammatory state comprise a multitude of chronic inflammatory disorders that significantly increase mortality. These can develop into common conditions like heart disease and cancer, which are all tightly linked to inflammation and obesity (2, 3). Other common chronic disorders are irritable bowel syndrome (IBS), which is characterized by gut dysbiosis and increased activation of immune cells (4) and inflammatory bowel disease (IBD), a group of diseases distinguished by chronic inflammation that includes Crohn’s disease and ulcerative colitis (5). Importantly, dysregulations in the intestinal microbiome and immune system also affect other organs, such as the brain, promoting the development of neurodegenerative disorders (6). Moreover, there is currently no cure for chronic diseases, although medical advances in disease management significantly decreased mortality in the past decade (7). Still, 3 out of 5 people die due to chronic inflammatory diseases worldwide (8). Not only does this constitute a huge health care and economic burden now, but prospects are not very optimistic with the rising trend of prevalence in chronic diseases (9). Hereby, one of the main difficulties of chronic inflammation is the silent progression in which it imperceptibly alters the metabolism and tissue homeostasis of the host, favoring the development of disease (10). One of the consequences can be obesity.

Obesity is considered to be the biggest global epidemic affecting individuals of every age (11). It is also strongly indicated to increase the risk of developing chronic diseases, such as depression, type 2 diabetes (T2D), cardiovascular disease (CVD), and cancer (12). All these are conditions that affect a tremendous amount of the world population and somewhat characterize modern society. The psychosocial costs caused by obesity and difficulties linked to associated comorbidities pose a significant economic burden. Prospects appear to be even worse as childhood obesity results in the same disorders with even earlier onset (13). Weight gain is not only a potential consequence of inflammation but commonly induces obesity-associated inflammation (14).

The most important factors of the vicious cycle between obesity and inflammation are alterations in the adipose tissue, interorgan cross-talk, and most importantly, obesity-induced lipopolysaccharide (LPS) leakage (15). The adipose tissue of obese people is characterized by an increased number or size of adipocytes (16). It is also associated with high infiltration rates of macrophages that are predominantly of the pro-inflammatory M1 phenotype (17). The resulting depletion of interleukin (IL)-10- and TGF-β-producing M2 macrophages does not only have consequences for immunoregulation but also for maintaining insulin sensitivity (18). Moreover, the secretion of proinflammatory cytokines and adipokines affect overall metabolic health, the endocrine and immune system via interorgan crosstalk (19). Thus, pro-inflammatory signals derived from adipose tissue can disrupt metabolic and immunologic homeostasis in other tissues as well, resulting in a systemic chronic inflammatory condition. Ultimately, continuous inflammation, metabolic dysfunction, and tissue damage strongly favor the development of serious diseases.

Although there are many ways in which adiposity and inflammation can be caused and promote the state of one another, gut-derived LPS is accounted to be one of the main causes, if not the main cause, of obesity-linked low-grade inflammation (20). In the case of gut homeostasis, a well-functioning epithelial barrier prevents bacterial components from leaving the gut and entering the bloodstream. Common observations made in obese individuals are alterations in the gut microbiome, increased gut barrier permeability, and higher LPS levels (15). Augmented LPS plasma levels have been linked to obesity-associated low-grade inflammation, as LPS can escape through a weakened gut barrier, travel through the bloodstream to other organs and trigger their local immune cells (21, 22). The pro-inflammatory stimuli in form of LPS prompts residing macrophages to polarize into M1 macrophages and further recruits circulating monocytes, which accelerates the disruptions in tissue homeostasis (15). Thus, serious impairments of the immune system and metabolism are believed to originate from the probably most important influencer of the immune system and systemic metabolism of the human organism – the gut microbiome.

The gut microbiota consists of a large number of microorganisms, which include bacteria, viruses, fungi, and archaea. The number of bacterial genes in the human body exceeds the host genome a hundred-fold, the gut microbiome being the largest and most diverse microbiome of the human body (23). It is dominated by the phyla *Firmicutes* (now referred to as *Bacillota* as determined by List of Prokaryotic names with Standing in Nomenclature (LPSN)), *Bacteroidetes*, *Proteobacteria* and *Actinobacteria* (now referred to as *Bacteroidota*, *Pseudomonadota* and *Actinomycetota*, respectively, according to LPSN), with *Firmicutes* and *Bacteroides* being the most abundant ones (24). Although it has been long known that bacteria play a crucial role in digestion and vitamin production, their essential role in regulating host metabolism and the immune system has become increasingly evident over the last few decades. For this reason, restoring gut homeostasis shows great potential in combating chronic inflammation and obesity. Gut commensals have a great share in maintaining gut homeostasis, as they impede pathogen growth via inducing colonization resistance and produce metabolites with antimicrobial activity (25). On top of that, bacterial metabolites also regulate host metabolism, such as tryptophan, which binds to the aryl hydrocarbon receptor (AHR), responsible for the transcription of phase I and II metabolizing enzymes (26). To date, certain bacterial residents were found to be crucial in promoting proper gut barrier function and an immunosuppressive environment. SCFA-producing bacteria play an important role in this context.

The three main SCFAs in the intestine are acetate, butyrate and propionate. SCFAs are mainly produced via fiber fermentation, although metabolic pathways of organic and amino acids can also result in SCFA production (27, 28). Although bacterial metabolism is highly strain-specific, generally, strains belonging to the phylum *Bacteroidetes* mainly produce acetate and propionate while butyrate is mainly produced by members of the *Firmicutes* phyla (27). Butyrate plays a particularly critical role in maintaining a healthy gut barrier as it is the primary energy source of intestinal epithelial cells. It also increases mucin production, and while it stimulates the proliferation of healthy colonocytes, its effect on cancerous colonocytes is the opposite (29). Thus SCFAs appear to have a sensitive role in regulating gut health. Apart from playing an important role as part of the physical barrier in innate immunity, SCFAs have a great impact on adaptive immunity, as well. For instance, SCFAs induce the differentiation and proliferation of regulatory T cells (Tregs), which are crucial for immunologic homeostasis (30, 31). SCFAs also affect B lymphocyte energy metabolism, shifting antibody production to immunoglobulin (Ig) A and modulating its activity towards commensal bacteria (32, 33). IgA is a key player in humoral mucosal immunity as it poses part of the first line of defense by binding bacterial toxins and fighting pathogens. Importantly, SFCAs also affect innate immune mechanisms, as butyrate and propionate were shown to inhibit mast cell activation (34). Thus, the pleiotropic role of SCFAs modulates both immune defenses and immunological non-reactiveness towards autoantigens, which makes them an essential factor in proper immunoregulation.

Furthermore, SCFAs also regulate host metabolism, as SCFAs can be recognized by several G protein-coupled receptors (GPRs), which are responsible for immune and metabolic regulation (35). This is crucial for limiting food intake and influencing glucose and lipid metabolism, as gut-derived SCFAs act on GPRs resulting in hormone production reaching the brain via the gut-brain axis (36). This way, SCFAs are able to impact appetite and insulin sensitivity. However, gut dysbiosis is not only associated with impairments in these regulations but also with LPS-induced metabolic endotoxemia further exacerbating energy intake and blood glucose levels (37). These mechanisms are also partly induced by epigenetic modifications (38).

Epigenetics modifications affect the transcription of genes without altering the genetic code, which are mainly conveyed by, but not limited to, DNA methylation, posttranslational histone modifications and different types of non-coding RNAs, such as microRNAs (miRNAs) and long non-coding RNAs (lncRNAs) (39). These epigenetic mechanisms globally regulate the expression of genes involved in metabolic and cellular inflammatory responses and are key in maintaining tissue homeostasis. Not only do epigenetics regulate these processes but epigenetic regulators are also strongly affected by metabolites, which is known as ‘metabolo-epigenetics’. SCFAs, particularly butyrate, has been shown to regulate immune cell differentiation and immunological pathways as well as a multitude of metabolic genes associated with obesity (40). This is highly relevant from a clinical point of view since metabolic and immunologic disruptions upon gut dysbiosis might be reversible. SCFA-induced epigenetic modifications might therefore be not merely a reasonable approach for preventing obesity and inflammation-associated pathophysiology but it could potentially reverse harmful developments initially caused by gut dysbiosis. Several murine and human studies show significant correlations between SCFAs and health status, but to translate these striking implications into clinical application, it is important to understand how obesity and inflammation can be practically and controllably modulated via epigenetic manipulation by SCFAs. Since SCFAs are implicated in several health-promoting processes, it would be more than reasonable to exploit their clinical potential by fully embracing their pleiotropic effects on overall health. This will also allow targeting of systemic issues of obesity and inflammation, which is urged by the global health situation.

The aim of this paper, therefore, is to explore the clinical potential of SCFAs, based on the most recent findings and current investigations in this field. To get a realistic picture of possible SCFA manipulation, the first part of this review focuses on the current knowledge about gut-altering factors affecting SCFA production. In the following, the complexity of the bidirectional interactions between metabolism and inflammation and the role of SCFAs in this will be thoroughly discussed. Hereby, both epigenetic-driven and independent activity of SCFAs is reviewed.

## 2 SCFA production in the gut

**Figure 1:**
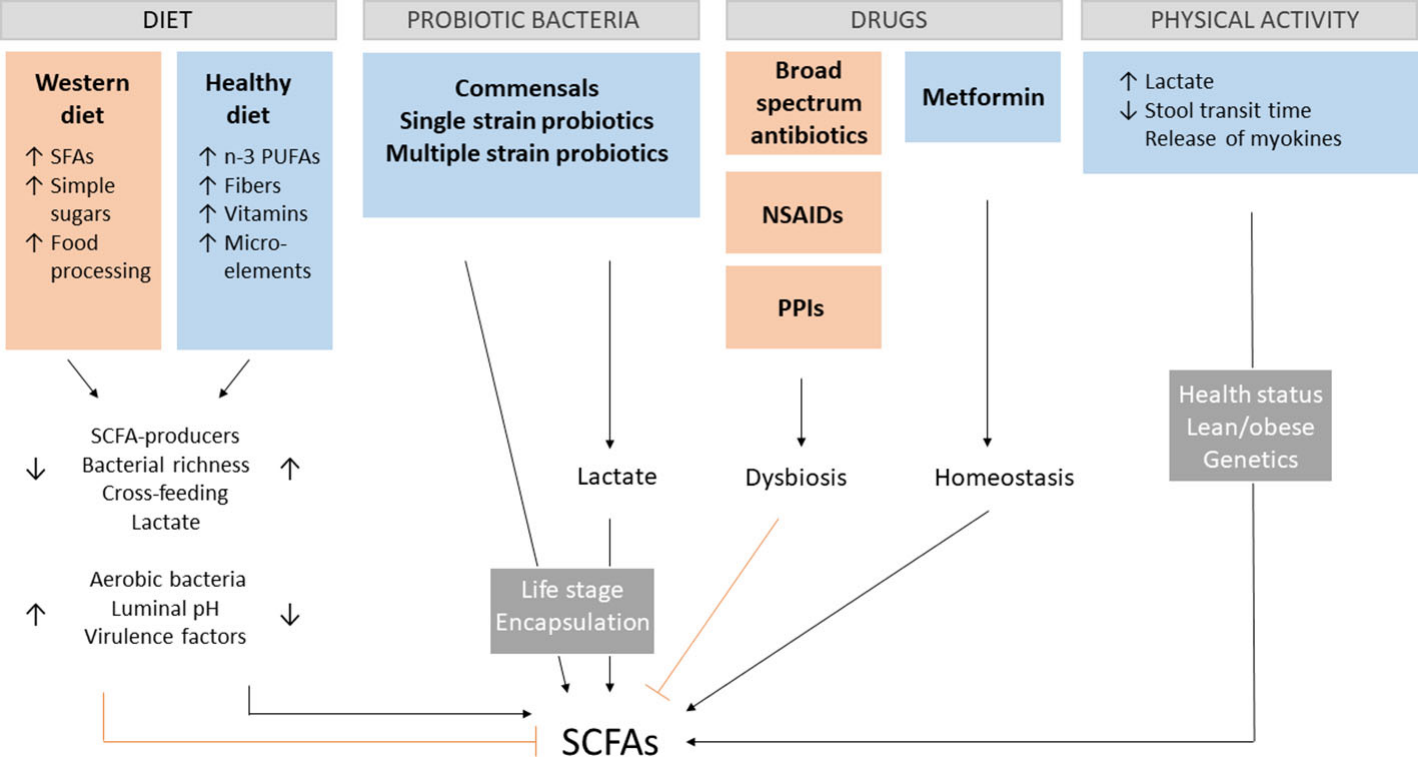
SCFA-influencing factors. Different aspects of the Western diet contribute to dysregulations in the gut environment, leading to the decrease of short-chain fatty acids (SCFAs). A positive impact on SCFA production has a diet rich in n-3 polyunsaturated fatty acids (PUFAs), fibers vitamins and microelements, by increasing the amount of SCFA-producing bacteria, overall bacterial richness, cross-feeding of species, lactate production and decrease the presence of unbeneficial aerobic bacteria, pH and expression of virulence factors. The opposite effect can be observed for the Western diet rich in saturated fatty acids (SFAs), simple sugars and processed food. The presence of key gut commensals and different kinds of probiotics enhance SCFA production directly or indirectly by lactate production with their effectiveness depending on the age of administration and encapsulation. Broad spectrum antibiotics, non-steroidal anti-inflammatory drugs (NSAIDs) and proton pump inhibitors (PPIs) favor gut dysbiosis associated with lower SCFAs levels. The opposite effect is the case for metformin. Physical activity modulates host health by crosstalk between skeletal muscle and the gut via myokines and lactate as well as direct physical stimulation. These effects depend on the health and metabolic state of the host, as well as genetics.

The prevalence of gut dysbiosis-associated diseases tremendously increased in the last decades, which can be attributed to the Western diet, sedentary lifestyle, and increased use of antibiotics (41). The importance of early microbial exposure and the danger of increasing sterility in developing an intact immune system has been already stressed in 1989 in Strachan’s hygiene hypothesis (42). Although this notion remains valid, the knowledge about particular influences on the gut microbiota and their impact on overall health becomes continuously deeper and better established. Since SCFA production depends both on the number of bacteria producing SCFAs and the availability of necessary nutritional substrates, it is important to understand the effect of different types of diets and how other factors can cause a shift in the gut microbiota composition. Although age, genetics, environmental and maternal factors are also involved in shaping the gut microbiome (43), this section will merely focus on the most important controllable factors altering SCFA production (**Figure 1**). This way, the clinical value of SCFA treatment can be realistically assessed.

### 2.1 Diet

Recent findings emphasize and specify the role of diet, which has a strong influence on the integrity of the intestinal barrier and gut microbiome composition, ultimately affecting SCFA levels. One of the characteristics of the Western Diet is high amounts of dietary fat. A high-fat diet (HFD) is associated with increased imbalances in the gut that also increase the risk for chronic inflammatory diseases (44). In this context, a diet rich in saturated fatty acids (SFAs) is associated with negative health manifestations whereas high amounts of unsaturated fatty acids, especially n-3 polyunsaturated fatty acids (PUFAs), exhibit beneficial health effects (44, 45). SFA interaction with GPRs and Toll-like receptors (TLRs) leads to the activation of pro-inflammatory mediators such as IL-1β and nuclear factor-kappa B (NFκ-B), a process prevented upon PUFA binding (45–47). In fact omega-3 fatty acids were shown to affect lipid metabolism and mediate anti-inflammatory effects via DNA methylation and miRNAs (48, 49), while the conventional Western diet negatively affects gene expression of inflammatory markers and metabolic genes due to epigenetic imprinting that can already take place *in utero* (50, 51).

Apart from the direct interaction with regulatory receptors, excess SFA intake has negative effects on the gut microbiome, reducing its diversity and richness and promoting the growth of facultative aerobic species which metabolize simple fats and sugars and at the same time decreases the proportion of beneficial anaerobes, including SCFA-producing *Akkermansia muciniphila* (52). Accordingly, the gut barrier-promoting and pH lowering effects of SCFAs decay as well. Worth noting, SFAs from different sources mediate different effects on gut microbiome diversity (52). Moreover, *A. muciniphila* and SCFAs were repeatedly shown to protect from HFD-induced obesity and metabolic anomalies, showing the important role of particular microbes in gut homeostasis (53–55), which is promoted by PUFAs (56, 57). Additionally, fibers have been also shown to reverse HFD-induced microbiota changes in diversity (58).

Dietary fibers were shown to counteract the negative effects of a Western Diet directly and by affecting the gut microbiome composition and thus metabolome and mediated epigenetic activity (59). For instance soluble fibers such as psyllium, were shown to hinder glucose and cholesterol absorption and bile salt reabsorption whilst improving the absorption of macro-and micronutrients (60). Fructooligosaccharides (FOS) and galactooligosaccharides (GOS) were shown to remedy gut dysbiosis and the associated gut barrier impairment and systemic inflammation in mice (61). Importantly, the mediated effect also depends on the amount and type of fiber administered and the presence of particular bacteria species. Regarding the amount of fiber, a diet rich in fiber exhibited an immediate effect on the gut microbiome diversity and metabolite production in humans (62), whereas low doses did not affect particular abundances in gut microbes (63, 64). When it comes to the different types of fibers, they can be categorized by fermentability, solubility and viscosity. Due to the fermentability of β-glucans, pectins and inulin, these fibers mainly promote systemic health via gut microbiota modulation (65) whereas non-fermentable fibers like psyllium, which has a high solubility and viscosity, independently improve digestion, glucose homeostasis and cholesterol levels (60). Due to the differently mediated benefits of various kinds of fibers, a plant-based diet comprising different types of dietary fibers is recommended. While complex carbohydrates exhibit various health-promoting effects, the opposite is true for simple carbohydrates.

A diet high in simple sugars, like glucose and fructose, is particularly problematic, as it decreases microbial diversity (66). It also increases the amount of aerobic *Proteobacteria* whilst decreasing *Bacteroidetes*, amongst which many SCFA-producers can be found (66). In the same study, high glucose and fructose diet ultimately led to gut barrier impairment resulting in metabolic endotoxemia, even without significant weight gain in mice. The consumption of artificial sweeteners was also shown to induce gut dysbiosis with metabolic consequences for the host (67). On the other hand, a diet low in polysaccharides has been shown to negatively affect bacterial abundance and richness (68). SCFA-producing bacteria are particularly affected due to the deprivation of their nutritional source. Moreover, the loss of one bacterial strain may affect the growth of others due to cross-feeding dependence, as is the case with *Ruminococcus gnavus* and Bifidobacteria, making SCFA-production by other strains possible via prior starch degradation or lactate production (69, 70). Furthermore, enhanced butyrate production upon starch intake was observed in individuals whose microbiota was enriched in *R. gnavus* and *Clostridium chartatabidum* (71), in turn eating behavior was shown to be influenced by the presence of SCFA-producing bacteria, as well (72). This suggests, that effective promotion of SCFA production via dietary intervention strongly depends on gut diversity and the presence of key bacteria species. In fact, prebiotic human intervention studies show strong variability amongst individuals, which only emphasized the issue of the complexity of individual genetic and gut microbiome predispositions (73). This highlights the great need for precise interventions, in which discrete dietary fiber structures and arabinoxylan show great potential and consistency among subjects (74, 75).

Apart from a poor-balanced diet, the Western diet is also characterized by various food additives and a high degree of processing. Recent evidence shows significant changes in gut microbiota upon processed food administration, with consequences in bacterial metabolism and weight gain (76). Artificial sweeteners and emulsifiers play an important role in altering host metabolism via the gut microbiota (67, 77). Not only does this affect metabolism, but it also promotes and exacerbates intestinal inflammation and leads to increased expression of virulence factors (78–80). These effects are believed to arise from gut dysbiosis, which favors the growth of pro-inflammatory species as opposed to immunomodulatory commensals. Processed food is not only enriched in chemical additives but certain food processing deprives the product of vitamins and microelements (81), which also affects SCFA production (82, 83).

Thus, the Western diet seems to decrease SCFA production on various levels, by deprivation of nutritional substrates and co-factors, which also play a role in epigenetic regulation (84), as well as by inducing gut dysbiosis and therefore promoting the growth of other species further exacerbating intestinal imbalance in the gut microbiome composition, metabolism and mucosal immune system. As an antidote, continuous intake of unprocessed food, rich in fibers, PUFAs, vitamins and microelements seems the most feasible to promote SCFA production via the diet.

### 2.2 Probiotics

As previously established, the presence and abundance of particular bacteria are crucial in enhancing SCFA production, due to their own ability to produce these compounds but also the effects they exhibit on other gut members. According to the Food and Agriculture Organization of the United Nations (FAO) and the World Health Organization (WHO), probiotics are ‘live microorganisms which when administered in adequate amounts confer a health benefit on the host’ (85). A probiotic may thereby consist of a single bacterial strain or contain multiple.

Regarding the former, several strains including *Lacticaseibacillus rhamnosus* GG (LGG), *Lactobacillus acidophilus* CRL 1014, *Lactobacillus gasseri* PA 16/8, *Ligilactobacillus salivarius* JCM 1230, *Ligilactobacillus agilis* JCM 1048, *Bifidobacterium longum* SP 07/3 and *Bifidobacterium bifidum* MF 20/5 were shown to produce SCFAs and lactic acid (86). In the study on the effect of oral consumption of *Lactiplantibacillus plantarum* P-8 (Lp-8) on human intestinal microbiota, and SCFAs of different aged adults (87), the increase in *Bifidobacterium* and other beneficial bacteria was found, whereas opportunistic pathogens decreased. Furthermore, a statistically significant increase in acetate and propionate levels in all age groups was observed. Thus single-strain-based probiotics seem to be able to competently promote gut homeostasis. Also commensal bacteria such as *Faecalibacterium prausnitzii* were shown to effectively promote gut diversity and SCFA production and by this mechanism, shows great potential in improving inflammatory and metabolic diseases (88–92). As previously mentioned, *A. muciniphila* administration can improve metabolic anomalies caused by gut dysbiosis (53, 54, 93). Thus single-strain-based probiotics seem to be able to competently promote gut homeostasis. Regarding multi-strain probiotics, SCFA production can be targeted on multiple levels. Co-cultures of lactic acid bacteria (LAB) were shown to produce SCFAs and stimulate mucus secretion of colonic epithelial cells (94). Moreover, a probiotic supplement (Symprove™), consisting of the four strains *L. acidophilus* NCIMB 30175, *L. plantarum* NCIMB 30173, *L. rhamnosus* NCIMB 30174 and *Enterococcus faecium* NCIMB 30176) was shown to competently increase SCFA production *in vitro*, as well (95). Not only were those strains shown to effectively colonize the colon but also to increase lactate concentrations. Lactate was further used by lactate-metabolizing bacteria resulting in enhanced SCFA production, particularly butyrate. In another study, a cocktail of several lactobacilli strains improved SCFA production, which dampened pathogen-induced pro-inflammatory signaling cascades (96). Moreover, another recent study revealed that more effective fiber fermentation occurs with co-cultures of strains from more than one genera, as in this case concerned *Lacticaseibacillus* and *Bifidobacterium* strains (97). This shows that the interaction between bacterial species can have a synergistic effect, emphasizing the important role of cross-feeding in the gut.

Nevertheless, less consistency is observed in human intervention studies. While some studies show significant differences in SCFA levels upon probiotic administration (98–100), others do not show any impact (101, 102). The results may differ between individuals due to genetic heterogeneity, differing health conditions, and microbiome composition, as well as due to different probiotic strains applied. The importance to obtain probiotics that are able to survive and colonize the gut can be seen by *ex vivo* human fecal microbiome culture systems, which are directly exposed to live probiotics and straightforwardly show the desired effects in SCFA production observed in mice (100). On the one hand, a more predictable tool to increase the translatability of mouse studies could be the investigating wild mice which have a more resembling microbial profile to humans (103). On the other hand, the fact that *in vivo* human studies do not demonstrate such strong translatability of mice studies, suggests that the amounts and viability of bacteria may be affected by several factors of the gastro-intestinal (GI) tract. Effective encapsulation and the ability of the probiotic to adhere to the gut epithelium play also an important role in this context (104–106). Recently, a lot of attention is directed towards bacterial exopolysaccharides (EPSs), which can be used as an effective carrier protecting from GI stressors, increasing muco-adhesion but also a synergistic compound to promote gut homeostasis (107–109). On another note, the robustness of the adult gut microbiome also needs to be acknowledged for effective clinical studies, as short-term interventions might be too weak to exhibit greater and long-lasting changes. The opposite can be observed in young infants, in which probiotic administration demonstrated improved clinical outcomes upon changes in the gut microbiome composition and metabolism (110–112). This can be explained by the critical time window of gut microbiome establishment, which makes it a sensitive target to manipulation (113, 114).

Thus, early prevention of gut dysbiosis, continuous long-term administration in adults and careful strain selection and encapsulation might be the most feasible approach to promote gut homeostasis and SCFA production via probiotics.

### 2.3 Antibiotics and non-antibiotic drugs

Apart from an altered diet, increasing sterility and intake of antibiotics and other pharmaceuticals are additional characteristics of modern Western Society. Narrow-spectrum antibiotics do not impact the gut microbiome composition as greatly as broad-spectrum antibiotics, which can have great consequences for intestinal microbiocenosis (115). These effects vary from the antibiotic administered, as well as from individual to individual. Nevertheless, broad-spectrum antibiotics such as ciprofloxacin and clindamycin revealed immediate decreases in SCFA-producing bacteria, where the restoration time to the initial gut microbiome in humans took up one month up to a year (116), while neomycin and ampicillin-induced gut dysbiosis in mice also affected epigenetic regulation of genes in the gut and liver (117).

SCFAs have an important share in mediating colonization resistance against pathogens and controlling the growth of opportunistic bacteria (118, 119). Their levels were shown to directly correlate with lower levels of the pathosymbiont *Escherichia coli in vitro* (120). Interestingly, in antibiotic-treated mice, increased levels of *Candida albicans* directly correlated to lower SCFA levels (121). In another study, similar observations were made and in addition, showed that the decreases in SCFAs also remained after the withdrawal of antibiotics (122). Moreover, long-term antibiotic-mediated SCFAs reduction stands in direct connection with disturbances in intestinal barrier integrity (123). Accordingly, an antibiotic-altered gut is more susceptible to recurring pathogenic infections in humans bearing metabolic consequences that promote obesity (124–126). Thus, antibiotic-induced lack of SCFA-mediated immunomodulatory and pro-homeostatic properties facilitates the colonization of pathogens and overgrowth of pathobionts, which increasingly occupy the niche otherwise dominated by beneficial commensals. The consequences are loss of SCFA-producing bacteria upon death by antibiotic exposure and are further aggravated by disadvantages in the competition of niche colonization by the pathogen-promotive environmental changes in the gut.

Other conventional non-antibiotic drugs such as proton pump inhibitors (PPIs) show similar effects as broad-spectrum antibiotics in altering the gut microbiome composition and immune system (127–129). On the other hand, metformin appears to mediate its beneficial metabolic effects against T2D by modulating the gut microbiome composition in a way that promotes SCFA-producing bacteria (130, 131). Apart from pharmaceuticals prescribed for particular health conditions, non-prescriptive non-steroidal anti-inflammatory drugs (NSAIDs) are not only widely used but also routinely. Routine consumption of aspirin, celecoxib and ibuprofen leaves a drug-specific signature in the gut microbiome, more so with stronger NSAIDs like ketoprofen, naproxen and ketorolac (132). In a recent study, short-term usage of celecoxib did not induce any significant changes in the gut microbiome whereas alterations in the metabolism of commensals decreased the production of butyrate (133).

Therefore it seems that both, the increased intake of antibiotics, PPIs and NSAIDs, with few exceptions, have their own contribution in inducing gut dysbiosis and consequential decrease in SCFAs.

### 2.4 Physical activity

As previously stated, a mainly sedentary lifestyle is a characteristic feature of Western Society. However, the various health benefits associated with physical activity become increasingly evident, as daily exercise decreases the risk of developing chronic metabolic and inflammatory disorders, as well as mental health issues (134, 135). Interestingly, its beneficial effects seem to be to some degree mediated by changes in the gut microbiome and can be also attributed to different epigenetic changes depending on the type and duration of the exercise (136).

Various studies present the benefits of exercise on metabolic health via an increase of SCFA-producing bacteria (137–140). A study comparing the effect of exercise in lean and obese individuals, however, did not show a significant shift in microbial metabolism for the latter (139). It is important to note though, that butyrate- and propionate-regulating genes decreased six weeks after ending the training period for obese individuals. In the same study, within obese individuals, butyrate and the activity of the butyrate-regulating gene BCoAT were associated with less body fat but more lean body mass and higher cardiorespiratory fitness. Thus, exercise-induced changes in the gut microbiota and SCFA-regulating gene expression appear to affect both groups, though differently. Obese individuals might therefore profit from SCFA-associated improvement in cardiorespiratory fitness, which may help in the adaptation process. Nevertheless, in some cases, such as insomnia, excess SCFAs are associated with negative health effects, whereas exercise was shown to beneficially regulate SCFA levels and sleep quality (141). Some studies also show that obesity can be associated with higher amounts of SCFAs as opposed to lean individuals and (142, 143). The same has been shown in mice, which upon receiving microbiota from exercise-trained donors gained more body mass (144). This might suggest that exercise promotes gut homeostasis accordingly to the needs of the individual, including proper balances in SCFA levels, regardless of whether a dysbiotic shift to scarce or excessive SCFA production has previously taken place. This also shows that health status, particularly obesity-related metabolic status, and genetics complicate gut microbiota manipulation via exercise, as it is not equally effective amongst individuals.

Moreover, short-term exercise was not proven to induce sustainable changes, which highlights the need for regular exercise to yield sustainable long-term effects regarding SCFA regulation (139).The individual response to exercise has been repeatedly reported to have a strong genetic base (145, 146). On top of that, different age groups and health conditions of study participants make it difficult to establish a clear relationship between SCFAs and exercise, not to mention the negligence of other confounding factors such as diet, which was shown to synergistically promote SFCA production (147). However, exercise also seems to have a diet-independent mechanism promoting SCFA production (148).

The standardization of SCFA measurements is another issue, as studies do not only differ in study designs (type of physical activity, duration, intensity, time without exercise), SCFA-measuring methods (type of SCFAs measured, gas chromatography, microbial profiling) but also in the sample type (plasma, feces) and sample processing (wet vs dry feces). Other aspects, such as SCFA absorption and interconversion by gut commensals, make it tricky to draw straightforward conclusions from fecal SCFA levels. Future research should therefore consider shedding more light on the underlying mechanism of increased/decreased SCFAs, with regard to the previously mentioned aspects. Thus, identifying SCFA-producing genes and ratios between acetate, propionate and butyrate may give additional insights into SCFA production/absorption (149, 150).

To better understand the varying observations in human studies, it is crucial to look at possible underlying mechanisms of exercise-induced changes in gut microbiota. One of the possible mechanisms is that movement physically stimulates gut microbiome function e.g. by reducing stool transit time (151). Furthermore, the underlying metabolic changes increase lactate production, which can be further metabolized to SCFAs by LAB (152). Another possible explanation could be the form of interorgan crosstalk between the gut and skeletal muscle. One of the mediators involved in this phenomenon are myokines, which are primarily cytokines that are released from skeletal muscle during exercise and exhibit anti-inflammatory properties that seem to have an impact on microbiota function in the intestine as well as systemically (153, 154). In turn, several microbial metabolites have been implicated in skeletal muscle metabolism. SCFAs are able to determine the efficacy of physical exercise by increasing lipolysis, promoting glucose uptake and insulin sensitivity in the skeletal muscle (153). SCFAs are not only an energetic substrate for colonic cells, but they also play an important role in muscle metabolism (155). Increased SCFA production seems to be a major player in mediating exercise-associated health benefits, as the donation of gut microbiomes from exercised mice was able to improve health outcomes in mice with chemically-induced colitis, mediated by the anti-inflammatory properties of SCFAs (144).

Thus, exercise-induced metabolic changes are able to influence the gut microbiome via bowel movement stimulation, myokine and lactate production. However, these effects depend on individual genetics, health status and metabolic profile. Longer-term intervention could possibly lead to more significant results in obese individuals, as increased expression of SCFA-regulating genes is associated with beneficial effects on cardiac and fat tissue metabolism. Thus, future studies should consider the long-term effects of exercise, possible confounding factors and multi-level SCFA analysis that pays special attention to SCFA absorption. More studies are also needed to define a healthy SCFA concentration range, which may differ between different age groups and health and obesity statuses. This also calls for standardizing SCFA-measuring methods.

## 3 SCFAs in obesity and inflammation

**Figure 2:**
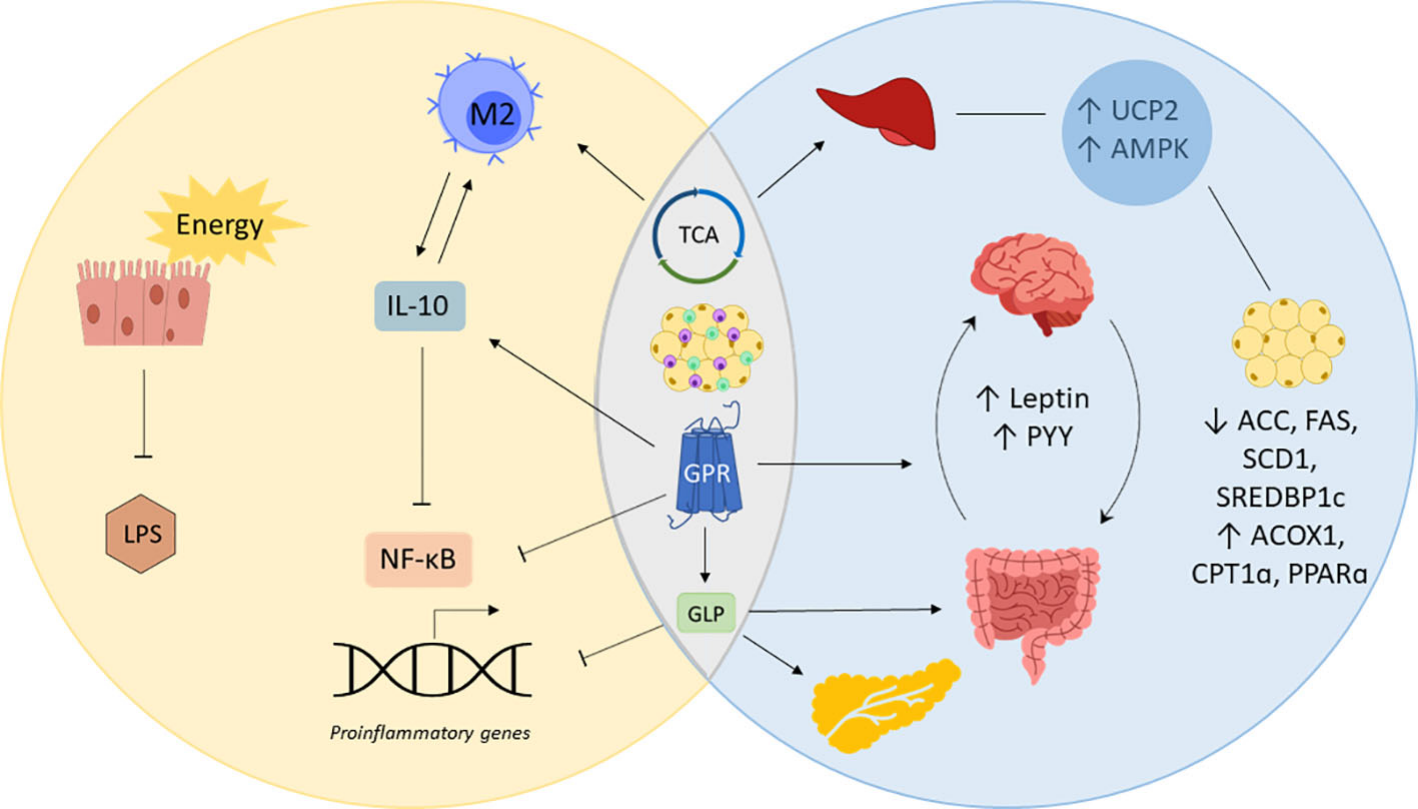
SCFAs in inflammation and obesity. Short-chain fatty acids (SCFAs) prevent inflammation by providing gut epithelial cells with energy enhancing cell renewal and gut barrier integrity, which decreases lipopolysaccharide (LPS) locally and systemically. SCFAs simultaneously regulate obesity and inflammation by acting on G-coupled protein receptors (GPRs), the tricarboxylic acid cycle (TCA) and immune cells of the adipose tissue. Posing a substrate for the TCA cycle and stimulating GPRs to produce interleukin (IL)-10, SCFAs promote the polarization of macrophages into IL-10 producing M2 macrophages, blocking nuclear factor kappa B (NF-κB)-induced expression of proinflammatory genes. Mitochondrial uncoupling protein 2 (UCP2) and AMP-activated protein kinase (AMPK) involved in liver and fat tissue metabolism are also regulated by SCFAs via the TCA. SCFAs decrease the expression of acetyl-CoA carboxylase (ACC), fatty acid synthase (FAS), sterol regulatory element binding protein-1c (SREBDP1c) while upregulating acyl-CoA oxidase 1 (ACOX1), carnitine palmitoyltransferase 1 (CPT1α) and peroxisome proliferator activated receptor α (PPARα). GPR-binding by SCFAs inhibits the transcription of proinflammatory genes and increases the release of appetite regulating hormones leptin and peptide YY (PYY). GPR-binding also causes the release of glucagon like peptide (GLP), which inhibits the expression of proinflammatory genes, promote the gut barrier and insulin sensitivity.

The unleashing role of SCFAs in overall systemic health also sheds special light to modulating issues directly linked to obesity and inflammation. As there are many mechanisms that particularly affect metabolic and inflammatory processes, it is important to understand the tight relationship between both states and what role gut-derived SCFAs play in it (**Figure 2**). For this purpose, evidence of the impact of SCFAs on obesity, inflammation, and related chronic conditions will be assessed in the following.

### 3.1 SCFAs in obesity

Currently, there is no cure for obesity, and only invasive interventions such as bariatric surgery have yielded significant success (156). This treatment is strongly indicated in cases of morbid obesity and metabolic diseases, however, as it is costly and invasive it is not a suitable option for all age groups and people bearing other health issues (157). Therefore, there is a great need for a safer and effective alternative, which brings attention to the clinical potential of SCFAs and their implications for obesity and the associated health conditions.

SCFAs play an important role in obesity and metabolic disease due to their pleiotropic and systemic health-promoting effects. Although some studies claim that SCFAs are increased in obese individuals and are hypothesized to be responsible for increased adiposity through excess energy (158, 159), other studies demonstrate the opposite (160–162). Moreover, recent research links probiotic-induced increased SCFA levels directly to weight loss (163–167), which also seem to exhibit their anti-obesity effect from early on via breastmilk (168, 169). Probiotics enhancing SCFA production decrease the expression of acetyl-CoA carboxylase (ACC), fatty acid synthetase (FAS), stearoyl CoA desaturase 1 (SCD1), and sterol regulatory element binding protein-1c (SREBP1c), all of which are involved in lipogenesis. Acetate, for instance, was shown to induce upregulation of acyl-CoA oxidase 1 (ACOX1), carnitine palmitoyltransferase 1 (CPT1α), and peroxisome proliferator activated receptor α (PPARα) which increased lipid oxidation and reduced adiposity and cholesterol levels (170, 171). Moreover, direct administration of SCFAs was shown to lower triglyceride levels in rats by decreasing ACC expression (172). Importantly, acetate and butyrate directly affect liver metabolism by posing metabolic substrates for the tricarboxylic acid cycle (TCA) (150). Other SCFA-mediated metabolic regulations of liver and adipose tissue concern the expression of mitochondrial uncoupling protein 2 (UCP2) via peroxisome proliferator-activated receptor gamma (PPARγ) (173). This increases the adenosine monophosphate (AMP)/adenosine triphosphate (ATP) ratio and aerobic metabolism in the liver and adipose tissue through AMP-activated protein kinase (AMPK)-signaling. Thus, Besten et al. conclude that SCFAs could serve as efficient PPARγ modulators that could improve metabolic syndrome.

SCFAs are also implicated in various metabolic processes responsible for proper energy expenditure and appetite regulation. They can function as ligands for GPR41 and GPR43 (FFAR3), which are receptors involved in metabolic pathways (171, 174). GPR41 activation causes the release of peptide YY (PYY), a gut hormone that regulates food intake and is involved in energy management. Moreover, leptin is also secreted upon SCFA stimulation of GPR41, which is a key regulator in appetite signaling (175). SCFA-binding to GPR4 triggers the release of glucagon-like peptide (GLP)-1 and GLP-2. GLP-2 promotes the integrity of the intestinal barrier by supporting epithelial cell renewal and tight junction proteins (176). GLP-1 has been shown to improve insulin sensitivity and inhibit glucagon secretion, which is why it is considered crucial in maintaining blood glucose homeostasis (177, 178). Furthermore, it plays an important role in controlling food intake, lipolysis and inflammation (179–181). It, therefore, poses a reasonable target for metabolic diseases and it is no surprise that altered SCFA levels are commonly observed in metabolically ill patients (182, 183).

### 3.2 SCFAs in chronic inflammation

Low-grade inflammation is a common feature in chronic conditions such as IBS, IBD, T2D, non-alcoholic fatty liver disease (NAFLD), CVD, and cancer. It is thought to originate from the systemic immune response triggered by LPS as a consequence of a leaky gut (20, 184). Since the immune system interacts with the gut microbiome, any dysregulations may lead to an endless loop of gut dysbiosis and systemic low-grade inflammation (185). A leaky gut can be caused by obesity or other factors causing gut dysbiosis, but it can also in turn induce obesity due to the associated systemic metabolic and inflammatory changes. Therefore it is hard to assess the primary cause of the vicious cycle between obesity and inflammation. Moreover, these are not entirely separate arrangements since metabolism determines the immunologic function, as well.

Chronic low-grade inflammation is marked by inflammatory cytokines such as tumor necrosis factor (TNF) α, IL-1, and IL-6 (37, 186). This immune response is activated upon endotoxin binding to lipopolysaccharide-binding protein (LPB). This complex can be bound to the CD14 receptor which activates the myeloid differentiation factor 88 (MyD88) and interleukin-1 receptor-associated kinase (IRAK)-1 signaling cascade upon activation of toll-like receptor (TLR) 4 (187). The fact that obesity is characterized by ongoing low-grade inflammation can be partly explained by the co-regulation of both states via GPRs. Not only are they involved in metabolic homeostasis but they also determine immune response. Since free fatty acids bind to these receptors, the absence of SCFAs necessary for the negative regulation of proinflammatory cytokines leads to the continuous expression of inflammatory mediators. In the presence of SCFAs on the other hand, nuclear factor kappa B (NF-κB)-mediated activation of TNFα, IL-6, and IL-12 is inhibited. Furthermore, SFCAs also increase IL-10 expression, which suppresses TNFα, macrophage inflammatory protein 2 (MIP-2), and IL-1β expression (188–190). Moreover, macrophages are thought to have a great contribution to low-grade inflammation in adipose tissue and systemically, and IL-10 has been shown to protect against endotoxemia and stimulate M2 polarization (191, 192). Moreover, TLR-mediated MyD88 activation and TNF signaling by M1 macrophages have further health consequences by impairing insulin signaling, which shows the tight relationship between inflammation and metabolic health (185). Moreover, the lack of SFCA gut barrier-promoting qualities facilitates the spread of gut-derived inflammatory molecules that can distribute systemically via the bloodstream. This way, LPS leakage through the epithelial barrier can also trigger inflammation in various tissues which might continue until gut homeostasis is reversed (193). If there is no resolution of inflammation, inflammatory mediators might amplify the response by recruitment of leukocytes which can bear systemic consequences in the long run.

Thus, obesity-induced gut dysbiosis that leads to metabolic endotoxemia lays the path for the development of inflammatory chronic disease. This results in aberrant interactions between the gut microbiome, host immune system and metabolism, leading to so-called metaflammation (185). Interestingly, in mice obesity-associated low-grade inflammation was mitigated by probiotics and correlated with SCFA levels (163).

### 3.3 Inflammation and obesity

The tight link between immunologic and metabolic function becomes evident in various organelles. For example, nutrient surplus in the endoplasmic reticulum (ER) can disrupt its function and trigger the unfolded protein response (UPR) that ultimately leads to the activation of various inflammatory pathways, such as c-Jun N-terminal kinase (JNK) and iκB kinase (IKK) (194). In consequence, insulin sensitivity and glucose homeostasis is disrupted, which will likely trigger again the UPR. Another organelle affected by obesity is mitochondria. Mitochondrial dysfunction caused by obesity results in increased reactive oxygen species that can further disrupt insulin-signaling and promote M1 polarization of macrophages and activate the inflammasome, which again leads to disruptions in metabolism and promotes obesity (195–198). Disruptions in nutritional or immune system homeostasis therefore further trigger pro-inflammatory responses and metabolic dysfunctions, closing the vicious cycle of promoting both states.

The crucial role of metabolism in the immune response becomes also clear in the case of innate immune cells. Immunological memory is not exclusively part of the adaptive immune system since innate immune cells also exhibit a certain degree of memory function, referred to as ‘trained immunity’. In an aim to elicit a faster and more effective response by the repeated encounter of a certain antigen, innate immune cells become intrinsically wired to increase the response to secondary stimuli, e.g. by LPS. This effect is partly mediated by increased sensitivity of pattern recognition receptors (PRRs) but also by a metabolic shift from oxidative phosphorylation to aerobic glycolysis, called the Warburg effect (199, 200). The latter is responsible for providing, for instance, macrophages with glycolysis- and broken tricarboxylic acid cycle (TCA)-derived substrates necessary to initiate a proinflammatory immune response. These include glycolytic enzymes, succinate, and citrate, but also glutamine and arginine catabolism aid M1 function (201, 202). Interestingly, low levels of LPS have the opposite effect, inducing tolerance (203). Therefore, gut homeostasis or dysbiosis differently impacts innate immune cell metabolism, which in turn directly dictates its function.

**Figure 3:**
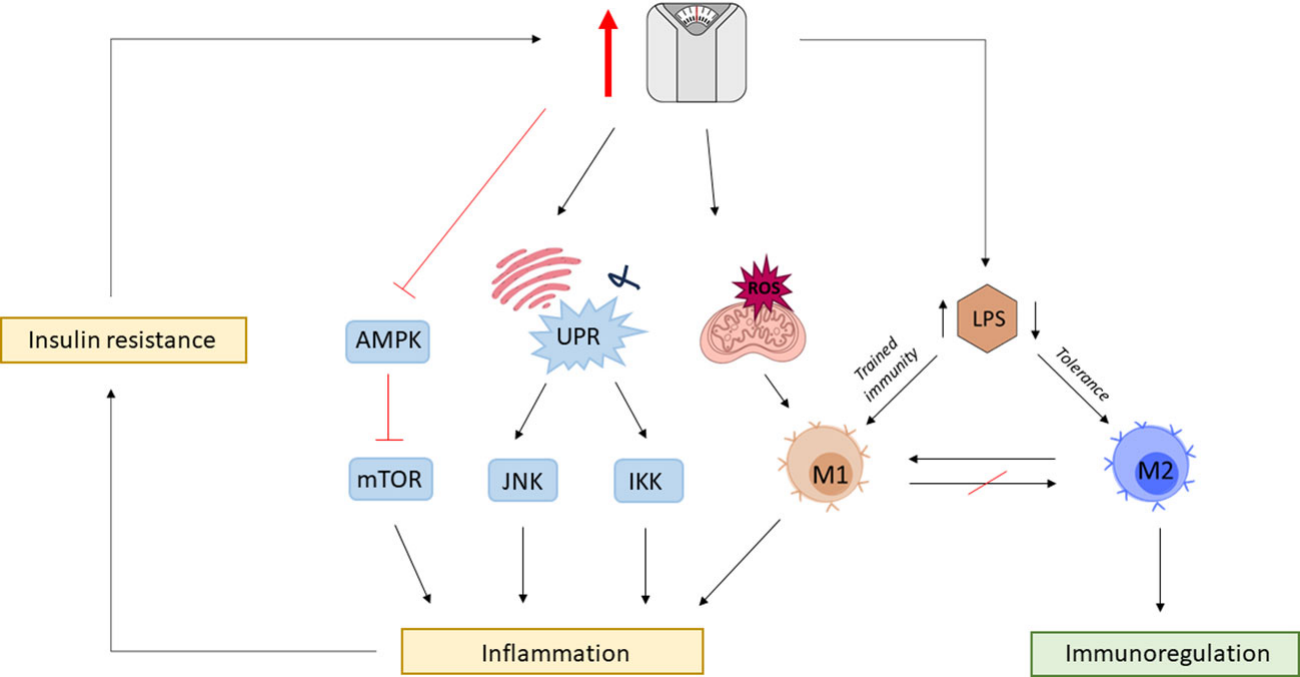
The vicious cycle of obesity and inflammation. Obesity is associated with gut dysbiosis and gut barrier impairment, leading to increased circulating levels of lipopolysaccharide (LPS). Chronic exposure to elevated LPS leaves a permanent phenotypic mark on macrophages, which disables otherwise mediated tissue homeostasis by M2 macrophages. Obesity is associated with mitochondrial dysfunction leading to the production of reactive oxygen species (ROS) promoting M1 polarization. Surplus nutrient intake also triggers mitochondria endoplasmic reticulum (ER) to activate the unfolding protein response (UPR) leading to the activation of c-Jun N-terminal kinase (JNK) and iκB kinase (IKK) proinflammatory signaling cascades. High nutrient availability also impedes the otherwise negative regulation of rapamycin (mTOR) by AMP-activated protein kinase (AMPK). Obesity-associated inflammation leads to metabolic changes resulting in insulin resistance further exacerbating the risk for obesity, closing the vicious cycle between immunologic and metabolic dysregulations.

Moreover, the mammalian target of the rapamycin (mTOR) pathway in innate immune cells is regulated by AMP kinase (AMPK), which in case of high nutrient availability, becomes deactivated (204). Due to mTOR diverse functions in cellular metabolism and homeostasis, dysregulation of mTOR signaling pathway has been implicated in various diseases (205). The loss of AMPK suppression of proinflammatory pathways can lead to hyperinflammation that can deteriorate underlying conditions such as atherosclerosis (206). Furthermore, it has been shown that the endotoxin-triggered proinflammatory phenotype of macrophages remains a long-lasting feature and that restoration of the anti-inflammatory M2 phenotype is straitened (199). Thus, the series of effects can be primarily triggered by LPS, having its roots in gut dysbiosis (**Figure 3**).

Interestingly, epigenetic mechanisms are responsible for innate immune memory with metabolic molecules being cofactors for epigenetic enzymes (207). Thus, LPS not only induces metabolic but also epigenetic changes within immune cells that prime them towards a pro-inflammatory phenotype. SCFAs on the other hand, show great potential in reversing those epigenetic dysregulations.

## 4 SCFA-epigenetic regulation

**Figure 4:**
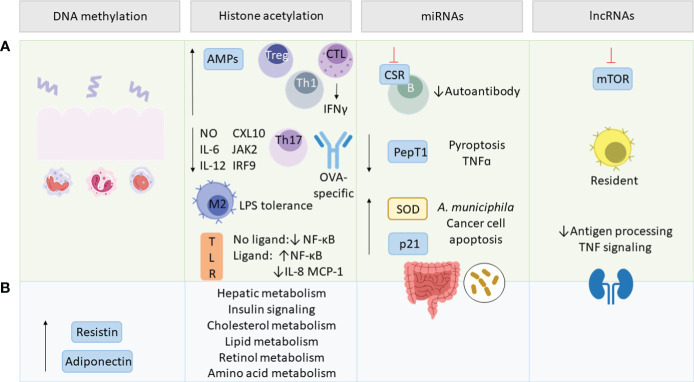
SCFA-mediated epigenetic regulations. Green section **(A)** addresses epigenetic effects in inflammation and blue section **(B)** in metabolism. SCFAs regulate intestinal epithelial cell (IEC)-related innate and adaptive immune functions, as well as resistin and adiponectin expression via DNA methylation. By histone deacetylase (HDAC) inhibition, SCFAs upregulate antimicrobial peptides (AMPs), T regulatory (Treg) and T helper (Th) cells and interferon γ (IFNγ) production by cytotoxic T lymphocytes (CTL). SCFAs mediate lipopolysaccharide (LPS) tolerance by M2 macrophage polarization and downregulate nitric oxide (NO), interleukin (IL)-6, IL-12, C-X-C motif chemokine ligand (CXL10), Janus kinase 2 (JAK2), interferon regulatory factor 9 (IRF9), Th17 and ovalbumin (OVA)-specific antibodies. They also modulate ligand-dependent responsiveness of toll-like receptor (TLR) leading to the up- or downregulation of NF-κB, IL-8 and monocyte chemotactic protein 1 (MCP-1) and affect various metabolic pathways. SCFA-modulated micro RNAs (miRNAs) act on class switch recombination (CSR) of B-cells, peptide transporter 1 (PepT1) and tumor necrosis factor α (TNFα) transcription, as well as pyroptosis. miRNAs upregulate superoxide dismutase (SOD), p21, cancer cell apoptosis, *Akkermansia muciniphila* abundance and impact the gut microbiome composition. SCFAs regulate long non-coding RNAs (lncRNAs) involved in rapamycin (mTOR) signaling, promoting resident macrophages and renal function via decreasing antigen processing and TNF signaling.

Epigenetic modifications contain a range of different mechanisms that alter DNA or DNA-associated proteins in a post-translational manner. This way the genetic code remains unaffected while protein expression can be regulated. SCFAs have been shown to modulate various pathways involved in inflammatory and metabolic regulations. Strikingly, epigenetic regulations initiated by the gut microbiome are not restricted to the gut but have been proven to impact other tissues as well (208). Epigenetic regulation via the gut microbiome can be mediated by providing cofactors for epigenetic enzymes in form of bacterial metabolites or by influencing the expression and activity of epigenetic enzymes and epigenetic pathways (209, 210). SCFAs play hereby a crucial role, since traveling from the intestinal lumen to other organs via the blood compartment they do not only maintain tissue homeostasis by binding to receptors but also by various epigenetic mechanisms (**Figure 4**). Under these fall modifications of DNA and chromatin-associated proteins, and non-coding RNAs (ncRNAs) (38).

### 4.1 DNA methylation

Epigenetic modifications of chromatin comprise both modification of DNA and of chromatin-associated proteins. Regarding DNA modification, the main epigenetic mechanism is the methylation or demethylation of genetic material. Methylated DNA is the result of additional methyl group attachment by DNA methyltransferases (DNMTs) to cytosine-phosphate-guanine (CpG) islands. Hereby, increased methylation of DNA is associated with a more compact chromatin structure and therefore decreased transcriptional activity, which can be beneficial in the context of obesity-associated genes and those involved in inflammatory responses (211).

DNA methylation by the microbiota plays an important role in intestinal homeostasis and balanced immune response. Microbiota-dependent DNA demethylation was shown to regulate the function of intestinal epithelial cells (IECs) and immune cells involved in innate and adaptive immune responses (212). Importantly, these epigenetic regulations affect the expression and responsiveness of TLR-4 upon LPS encounter and promote the proliferation of intestinal Tregs and natural killer lymphocytes (213–215). Moreover, IBD-associated mucosal inflammation is associated with aberrant DNA methylation whereas SCFAs exhibit anti-inflammatory effects in IBD via IECs-induced changes in the transcription of inflammatory markers (216–218). On the other hand, gut dysbiosis, the presence of bacterial pathogens or LPS affect epigenetic mechanisms connected to inflammation and metabolism by acting on host DNMTs or by producing the enzyme itself (219–222).

Mouse studies also reveal the direct role of SCFAs in the epigenetic regulation of obesity-associated proteins (223). Supplementation of acetate, propionate, and butyrate in mice was able to reverse the reduced expression of adiponectin and resistin in adipose tissue of HFD-fed mice by lowering the expression of DNA methyltransferases and methyl-CpG-binding domain protein 2 (MBD2). Since adiponectin is also considered to have insulin sensitivity-promoting, anti-inflammatory, and anti-atherogenic properties, there is great interest in increasing its expression in obesity and chronic inflammatory conditions (224). On the other hand, resistin levels above the norm are associated with obesity and T2D, whereas normal levels do not exhibit harmful effects (225, 226). Human studies also confirm disrupted DNA methylation in the adipose tissue of metabolically ill or overweight subjects and point to the probable role of SCFA due to the association of methylation status and abundance of SCFA-producing bacteria (227, 228). However, in yet another mouse study, antibiotic-induced changes in the gut microbiome lead to weight loss and increased adiponectin and resistin in an SCFA-independent manner (229).

Moreover, DNA methylation can be also influenced by other bacterial metabolites serving as cofactors of epigenetic enzymes, such as B group vitamins and polyamines (230–232). Into the bargain, the impact of SCFAs on host health appears to be distinct between lean and obese individuals. While SCFAs seem to prevent lean individuals from obesity and metabolic disturbances, in obese individuals, SCFA-mediated DNA methylation makes them more prone to develop T2D (233). Moreover, epigenetic alterations induced during early gut microbiome development were shown to have a great impact on individual predispositions regarding gut microbiome composition and metabolic and immunological profile (234–236). Importantly, early intervention in gut microbiome-induced epigenetic mechanisms via maternal probiotic intake shows great potential in modulating short- and long-term epigenetic patterns that beneficially regulate health (237, 238).

### 4.2 Histone modification

Amongst all epigenetic modifications, histone acetylation has been most thoroughly studied and the role of SCFAs in this context is also better understood. Histone acetylation is regulated by two catalytic enzymes – histone acetyltransferase (HAT) and histone deacetylase (HDAC). The latter is responsible for deacetylation and poses the main target of SCFAs. As previously mentioned, SCFAs influence the metabolism and increase the availability of metabolic substrates needed for histone acetylation, such as acetyl-CoA (239). Other post-translational histone modifications mediated by different SCFAs are methylation, ethylation, crotonylation, propionilation, butyrylation and hydroxybutyrylation, but these mechanisms just begin to be understood (239–244).

Similarly as DNA methylation, histone methylation of immunoregulatory and antioxidant genes is distinct in IBD patients and modulated by the gut microbiota (245). However, histone acetylation/deacetylation is a better studied process of epigenetic histone modification. Bacterial metabolites were shown to affect the acetylation status of genes from IECs and macrophages, modulating antimicrobial immune response (246, 247). Amongst all SCFAs, butyrate appears to be the strongest HDAC inhibitor and by this was shown to regulate the immune system and metabolism systemically. Mice and *in vitro* experiments show that the HDAC-inhibiting activity of butyrate increases antimicrobial activity of macrophages while suppressing nitric oxide (NO) expression and thus greater flare up of inflammation (247). In another study, antimicrobial peptides (AMPs) were shown to be upregulated via butyrate-induced HDAC inhibition, enhancing targeted pathogen elimination (248). Importantly, HDAC inhibition by butyrate was shown to induce the anti-inflammatory and resolutive macrophage M2 phenotype and generate tolerance towards LPS by downregulating NO, IL-6 and IL-12 (249, 250). Similarly, other studies show selective histone deacetylation of inflammatory genes during LPS-induced immune response in epithelial cells, suppressing inflammatory pathways induced by C-X-C motif chemokine ligand 10 (CXL10), Janus kinase 2 (JAK2) and interferon regulatory factor 9 (IRF9) (251). Another study points to the regulatory effect of SCFAs on NF-ĸB expression, dependent on the absence or presence of TLR ligands (252). In the latter case, SCFAs enhanced NF-ĸB expression during TLR stimulation, but downregulated the chemotactic cytokines IL-8 and monocyte chemotactic protein 1 (MCP-1). Thus SCFA-mediated histone modifications affecting the immune system enhances defenses against pathogens whilst sparing the intestine from excessive inflammation – relevant in counteracting chronic low-grade inflammation.

Histone modifications also play an important role in the immunoregulation of adaptive immune cells. SCFAs were shown to influence genes responsible for class switching in B cells via HDAC inhibition, reducing affinity to the ovalbumin (OVA) allergen (253). In addition to GPR signaling, HDAC inhibition at the forkhead box P3 (FOXP3) locus promotes differentiation and proliferation of Tregs, a key mediator of tolerance and homeostasis (30, 254–256). Interestingly, SFCAs also exhibit synergistic effects on intestinal immunity via GPR stimulation and HDAC inhibition in T cells and ILCs, resulting in increased expression of the gut barrier-promoting IL-22 (257). Moreover, butyrate was shown to decrease T helper cell (Th) 17 expression and increase Th1 via histone acetylation (258, 259). On top of that, SCFA enhance interferon γ (IFNγ) expression in cytotoxic T lymphocytes (CTLs) via HDAC inhibition, crucial for mediating anti-tumor effects (260).

SCFAs were also shown to affect histone acetylation and methylation beyond the intestine, as they epigenetically regulate gene expression in the liver, kidney and fat tissue (208, 261). Mice fed a Western diet showed decreased levels of SCFA resulting in altered histone modifications ultimately regulating hepatic metabolism via PPAR signaling, genes involved in insulin and cholesterol pathways, as well as genes related to adaptive immunity, lipid, retinol, and amino acid metabolism (208). Interestingly, SCFA supplementation of acetate, propionate, and butyrate was able to reverse the epigenetic phenotype to such a degree that it resembled the one from conventionally raised mice.

Although the sensitive role of SCFAs in modulating the immune system and metabolism becomes clear in the case of histone modification, the effect of SCFAs is not entirely straightforward. Different bacterial metabolites can be antagonistic and can act differently on HDACs. Although it is generally thought that SCFAs act beneficially as HDAC inhibitors, it was shown that butyrate can interfere with IBD-counteracting inositol-1,4,5-triphopshate (INSP_3_)-mediated histone deacetylation (246). Moreover, Kespohl et al. suggest that the beneficial action of butyrate might be dependent on its concentration and immunological predispositions (262). For safe and effective SCFA-mediated epigenetic regulation, It might be necessary to formulate a personalized approach towards each individual with regard to their metabolic status, immunologic and microbiome profile.

### 4.3 Non-coding RNAs

Important regulators of gene expression are ncRNAs, which are RNA molecules that are not coding for a protein and do not undergo translation. Besides the housekeeping ncRNAs like transfer RNA (tRNA) or ribosomal RNA (rRNA), there is a range of regulatory RNAs. Regarding the latter, miRNAs are the most abundant ncRNAs and also the best studied. miRNAs have around 20 nucleotides and regulate gene expression by binding to complementary target messenger RNA (mRNA), which suppresses translation either by degradation or silencing of the bound mRNA fragment.

Studies reveal a correlational relationship between bacteria, SCFAs and miRNAs regulating inflammatory processes in the intestine as well as in other organs (263–267). The gut microbiota was also shown to downregulate proinflammatory cytokines and the transport of proinflammatory bacterial peptides by peptide transporter 1 (PepT1) via miRNA upregulation (268–270). Moreover, probiotic strains producing SCFAs and lactate were shown to modulate miRNA expression resulting in anti-inflammatory and gut barrier-promoting effects (271). What is more, high doses of butyrate and propionate were shown to directly modulate B cell-intrinsic mechanisms of class switch recombination (CSR) by targeting associated genes via the upregulation of miRNAs (253). Not only did this limit autoantibody production in mice but the manner in which miRNAs were upregulated was the result of HDAC inhibition of miRNA-encoding genes and thus was found to be a form of epigenetic crosstalk. The same has been shown for DNA methylation which can affect miRNA expression (272). Interestingly, certain miRNAs can affect DNA methylation by targeting DNMTs and ten-eleven translocation enzymes (TETs) acting as methylation erasers (273–275) and this way can also impact the expression of other miRNAs (276).

In addition, miRNAs also regulate the gut microbiome composition and SCFA receptors showing the bidirectional regulation of SCFAs and miRNAs (277–279). The same goes for diet- and host-derived miRNAs, which were shown to affect gut microbiota (280, 281). Moreover, several epigenetic mechanisms can work in synergism as has been shown for SCFA-mediated regulation of the expression of cyclin-dependent kinase inhibitor p21 in human colonic cancer, which can be enhanced by miRNA degradation and histone acetylation (282, 283). Synergistic regulation was also found for several miRNAs and butyrate regarding colon cancer cell apoptosis (284). Other bacterial metabolites, such as tryptophan, were shown to control adiposity and insulin sensitivity through miR-181 expression (285). Butyrate, on the other hand, was shown to dampen inflammatory dysregulations in T2D patients by decreasing pyroptosis and TNF-α levels whilst increasing superoxide dismutase (SOD) activity and *A. muciniphila* abundance (286, 287).

lncRNAs are RNA fragments with up to 200 nucleotides and can regulate gene expression by various posttranslational protein modifications such as protein phosphorylation, ubiquitination and acetylation, as well as DNA methylation (288). They can also affect miRNA expression and vice versa (288, 289). The gut microbiome was shown to regulate lncRNAs within and beyond the intestine (290, 291). Hereby, bacterial components such as LPS negatively affect the host transcriptome and proteome, promoting inflammatory processes and cancer development (292–294). On the other hand, SCFAs were shown to suppress mTOR signaling and induce macrophage differentiation into resident macrophages (295, 296). LncRNA also play an important role in obesity, regarding energy expenditure, appetite regulation, insulin sensitivity and other obesity-associated inflammatory conditions (291, 297–300). SCFAs were hereby shown to regulate lncRNAs preventing renal dysfunction of diabetic nephropathic mice by e.g. decreasing antigen-processing and TNF signaling (301).

Recent studies also hint at the involvement of the gut microbiome in regulating other non-coding RNAs, such as small nuclear RNAs (snoRNAs), piwi-interacting RNAs (piRNAs) and circular RNAs (circRNAs) (302–304). However, the underlying mechanisms and possible role of SCFAs in this context remain to be discovered.

## 5 Conclusion

Overall, the epigenetic activity of SCFAs shows great potential in reversing the metabolic and immunological defects caused by metabolic endotoxemia and thus break the vicious cycle of obesity and inflammation. It is important to keep in mind, that the treatment in adult, obese and chronically ill individuals requires a long intervention time and personalized approach to overcome the prevailing individual metabolic and immunological predispositions. In order to find an effective strategy in humans, a synbiotic containing fibers and beneficial bacterial key species but also enriched in other SCFA-promoting substances and cofactors such as lactate, B group vitamins and microelements could prove efficacious. Nevertheless, diet and lifestyle should be an additional angle to counteract obesity-associated low-grade inflammation, as not only does it influence SCFA production but also conveys distinct health-promoting effects that can have a synergistic effect, which is desirable for the complex issues of obesity and inflammation. This might give a better chance to reestablish healthy interorgan crosstalk of myokines, cytokines and host miRNAs derived from adipose tissue, skeletal muscle and liver, all of which also influences the gut microbiota and its regulative function. More long-term and standardized research is still needed to better understand how to effectively modulate metabolism and the immune system via SCFA-mediated epigenetic modification. However, in the long run, SCFA treatment could become an accessible standard therapy for both prevention and treatment of obesity and inflammation.

## Author contributions

JK: Conceptualization, Visualization, Writing – original draft, Writing – review & editing. MK: Funding acquisition, Supervision, Writing – review & editing.
